# Violence Against Healers in Italy: A Medico-Legal Inquiry into Patient Aggression

**DOI:** 10.3390/healthcare13161947

**Published:** 2025-08-08

**Authors:** Paolo Bailo, Filippo Gibelli, Marilyn Cennamo, Giuliano Pesel, Emerenziana Basello, Tommaso Spasari, Giovanna Ricci

**Affiliations:** 1 Section of Legal Medicine, School of Law, University of Camerino, 62032 Camerino, Italy; paolo.bailo@unicam.it (P.B.); marilyn.cennamo@studenti.unicam.it (M.C.); giovanna.ricci@unicam.it (G.R.); 2 Department of Medical, Surgical and Advanced Technologies “G.F. Ingrassia”, University of Catania, 95121 Catania, Italy; 3 Nursing Degree Course, University of Trieste, 34127 Trieste, Italy; 4 Section of Occupational and Legal Medicine and BioLaw, Niccolò Cusano University of Rome, 00166 Rome, Italy

**Keywords:** workplace violence, healthcare workers, public health governance, Italian health policy, occupational safety

## Abstract

In recent years, Italy has experienced a significant increase in violence against healthcare workers, mirroring a global trend. Manifesting as verbal, physical, psychological, and material aggression, this phenomenon endangers both personnel safety and the foundational principles of the National Health Service (SSN) as outlined in Article 32 of the Italian Constitution. The escalation—most acute in emergency departments, psychiatric units, inpatient wards, and community services—affects a broad spectrum of professionals, compromising care quality and institutional integrity. Data from the FNOMCeO-CENSIS Report 2023–2024 reveal over 18,000 reported incidents in 2024, with verbal assaults disproportionately affecting female nursing staff. The COVID-19 pandemic further exacerbated systemic vulnerabilities, heightening user dissatisfaction and psychological strain among healthcare providers. In response, legislative actions—such as Law No. 113/2020 and Decree-Law No. 137/2024—aim to strengthen prevention, monitoring, and penal measures. This article examines legal, institutional, and organizational responses, including on-the-ground and hospital-based strategies to mitigate violence. Adopting a multidisciplinary perspective, it analyzes recent policy developments, regional dynamics, and victim-perpetrator profiles, arguing that safeguarding healthcare environments is both a public security priority and an ethical imperative essential to preserving the dignity of care work and the resilience of the health system.

## 1. Introduction

In recent years, violence against healthcare professionals has emerged as a critical global concern, affecting the safety, dignity, and sustainability of health systems worldwide [1,2,3]. According to data from the World Health Organization (WHO) and the International Labour Organization (ILO), healthcare workers are involved in approximately 50% of all workplace violence incidents, a proportion that underscores their exceptional vulnerability [4,5]. Across the globe, reported cases of violence have surged: assaults on healthcare workers have increased by 39% globally, with specific spikes observed in the United States (+40%), the United Kingdom (+35%), and Europe (+32%) [6]. The Middle East and Africa continue to record the highest rates of physical assaults against doctors and nurses. In the United States, healthcare workers are five times more likely to experience workplace violence than workers in other sectors, accounting for 70% of all reported nonfatal incidents [7]. Between 2011 and 2013, 47 shootings occurred in U.S. healthcare facilities, 60% of which took place in trauma centers and 78% of which involved targeted victims. Canada reports similarly alarming data, with 60% of emergency department nurses having experienced violence and expressing fear in patient interactions. European data reflect a comparable trend: the European Working Conditions Survey (EWCS) found that 16% of workers reported threats and 15% experienced actual violence within a year.

The NEXT (Nurses’ Exit Study) project, involving over 38,000 nurses across ten countries, identified France (39%), the United Kingdom (29%), and Germany as having particularly high prevalence rates, with lower figures in Norway (9%) and the Netherlands (10%). Emergency departments and psychiatric units consistently emerge as the most violence-prone settings. Despite these alarming statistics, the lack of harmonized international data collection frameworks and the historical underrepresentation of physicians in studies remain significant barriers to comprehensive understanding and policy development. In response, organizations such as the WHO, ILO, International Council of Nurses (ICN), and Public Services International (PSI) have developed standardized guidelines and methodologies to document and mitigate workplace violence in both emergency and non-emergency healthcare settings.

The Italian context reflects many of these global dynamics while presenting its own unique challenges. Violence against healthcare personnel in Italy has intensified in recent decades, affecting not only frontline professionals but also the foundational principles of the SSN (Servizio Sanitario Nazionale—National Health System), grounded in Article 32 of the Italian Constitution and the values of universality, equity, and solidarity. Episodes of verbal, physical, psychological, and material aggression are increasingly reported in emergency rooms, psychiatric wards, inpatient units, and community health services. A wide range of professionals—physicians, nurses, auxiliary staff, volunteers, and even security personnel—are subject to such acts, which compromise not only their physical safety but also their psychological resilience, professional integrity, and job satisfaction. These attacks are often triggered by patient dissatisfaction, prolonged waiting times, negative clinical outcomes, systemic inefficiencies, or heightened emotional states, especially in acute care settings. The COVID-19 pandemic has further exacerbated these tensions, revealing pre-existing structural fragilities and amplifying the pressure on already overburdened healthcare staff. According to the 2023–2024 FNOMCeO-CENSIS (FNOMCeO: Federazione Nazionale degli Ordini dei Medici Chirurghi e degli Odontoiatri—National Federation of the Orders of Surgeons and Dentists; CENSIS: Centro Studi Investimenti Sociali—Center for Social Investment Studies) Report, more than 18,000 healthcare workers were victims of violence in Italy, with an average of at least 116 incidents reported per local health authority in 2024 alone. Over 70% of these involved verbal aggression—often underestimated but capable of causing long-lasting psychological harm. The consequences are both direct (physical injuries, trauma, absenteeism, infrastructure damage) and indirect (decreased work morale, increased turnover, and erosion of public trust in healthcare institutions).

Italy’s institutional response has evolved progressively. Law No. 113/2020, enacted during the early stages of the COVID-19 pandemic amid widespread public admiration for healthcare workers, established the ONSEPS (Osservatorio Nazionale sulla Sicurezza degli Esercenti le Professioni Sanitarie—National Observatory on the Safety of Health and Social Care Professionals), tasked with monitoring violence, promoting prevention, and coordinating institutional responses. However, the recurrence of serious incidents—such as the double assault at the Foggia hospital in 2024—prompted the government to adopt more stringent measures through Decree-Law No. 137/2024. This decree introduced tougher criminal penalties for assaults on healthcare workers, revised the prosecutability of such offences, and mandated the restructuring of surveillance and prevention protocols within healthcare facilities. These legislative efforts reflect a growing recognition that violence against healthcare personnel is not only a matter of workplace safety but a profound ethical and civic issue that calls into question the very resilience and humanity of our public health systems. Addressing this phenomenon requires integrated, multidisciplinary strategies—legal, organizational, and cultural—that protect workers, support victims, and reaffirm the social value of care work.

This article proceeds in three parts. First, it presents an overview of national data on the incidence and characteristics of violence against healthcare personnel in Italy. Second, it offers a critical analysis of the legal and institutional responses implemented at both national and regional levels. Third, it outlines policy and practice recommendations to strengthen prevention, protection, and prosecution strategies. Although the focus is on Italy, this case offers broader relevance due to the country’s rapidly escalating incident rate, its distinctive medico-legal and institutional framework, and its potential to inform similar debates across Europe.

A comparison with international legal frameworks reveals that Italy’s response aligns with, yet also differs from, measures adopted elsewhere. For example, France has implemented specific criminal provisions to protect emergency medical personnel (Loi n° 2021-502), while the UK’s Assaults on Emergency Workers (Offences) Act 2018 increases penalties for attacks on healthcare staff. In the United States, several states have passed workplace violence prevention laws that mandate safety plans in hospitals. Italy’s integration of both punitive and preventive tools—such as the National Observatory and the Health DASPO—positions it as a unique case study in the European context. A comparative summary is presented in Table 1.

## 2. The Legal Evolution of Healthcare Worker Protection: From Decree-Law No. 137/2024 to Law No. 171/2024

In Italy, the right to health is enshrined in Article 32 of the Constitution [8] and is guaranteed through the SSN, established by Law No. 833/1978 [9]. Grounded in the principles of universality, equality, and equity, the SSN ensures access to healthcare for all citizens, financed through general taxation [10,11,12]. Key milestones in its development include the 1968 hospital reform [13], the Basaglia Law [14], and the constitutional reform of Title V in 2001, which enhanced the role of regional governments [15]. The system guarantees the Essential Levels of Care (LEAs), fostering a solidarity-based and democratic model of public healthcare.

Undoubtedly, the establishment of the SSN marked a significant democratic advancement in the realization of both the right to care and the right to health. It represents a model of development that supports citizens by providing healthcare through the collective effort of public financing, making health accessible to all—a shining example of solidaristic care [16,17]. Since the conclusion of the COVID-19 emergency phase in 2022, a troubling increase in violent episodes targeting health care personnel—often accompanied by the vandalism and destruction of medical facilities—has raised significant concern among institutional actors and the public alike. These assaults, frequently taking place in emergency departments, have involved individuals who initially sought medical assistance, only to direct their frustration and aggression toward medical staff and hospital infrastructure [18].

The rise in violence against healthcare professionals, particularly in emergency departments, led the government to adopt stricter measures to protect personnel and public health facilities. Law No. 56 of 26 May 2023, converting Legislative Decree No. 34/2023 [19], amended Article 583-quater of the Criminal Code. It now penalizes with imprisonment from two to five years any injury inflicted on healthcare or social-health workers, or on those in related support roles, during or because of their duties. For serious and very serious injuries, penalties rise to four–ten and eight–sixteen years, respectively, aligning healthcare workers with public officials in terms of legal protection. Additionally, police commissioners may now establish permanent State Police posts in hospitals with emergency departments to ensure safety. The 2023 reform also removed the previous threshold of injury severity, making all forms of violence—minor, serious, or very serious—against healthcare personnel prosecutable ex officio.

The further escalation of this phenomenon compelled legislative intervention, culminating in the promulgation of Law of 18 November 2024, No. 171, which converted into law Decree-Law of 1 October 2024, No. 137, titled “Urgent measures to counteract violence against health, social, and auxiliary workers engaged in care and treatment, as well as damage to property intended for health care” [20]. This legislative measure aims to reinforce the legal protection of health care professionals and to address, with greater rigor, the growing wave of hostility encountered within clinical settings. Compared to the original decree-law, the conversion into law expanded the scope of protection and the range of applicable sanctions. Specifically, the provisions on injuries inflicted on health and social-health professionals during the exercise of their duties were extended to include individuals performing complementary security services. Moreover, the law introduced a new criminal offence concerning the damage of property designated for health or social-health purposes—particularly when such acts are committed within, or in the immediate surroundings of, healthcare and residential facilities, and are accompanied by violence or threats or occur concurrently with personal injury to healthcare personnel.

The new regulatory framework stipulates imprisonment ranging from two to five years for those who cause injury to healthcare and auxiliary personnel in the exercise of their duties [21]. These sanctions align with and expand upon those already provided by Law No. 113 of 14 August 2020, which had previously introduced dedicated safeguards for the safety of healthcare professionals [22]. Under the latter, penalties for serious injuries range from four to ten years of imprisonment, and from eight to sixteen years for very serious injuries. Such crimes, under the new framework, are prosecutable ex officio, thereby eliminating the need for a formal complaint from the victim.

*Law No. 171/2024* also amends Article 635 of the Penal Code by introducing a new paragraph that provides for imprisonment from one to five years and a fine of up to 10,000 euros for anyone who, using violence or threats, damages or renders unserviceable movable or immovable property located in or designated for health or social-health services, whether public or private. Furthermore, the law introduces an aggravated form of the offence, with an increase in penalties of up to one-third when the act is perpetrated by multiple individuals acting in concert [23].

In a significant innovation, the law establishes both mandatory and deferred arrest in flagrante delicto for specific offences, including bodily harm against healthcare personnel and damage to health-related property. The Code of Criminal Procedure has been accordingly amended to allow for deferred arrest in cases of non-negligent crimes committed within or around healthcare or social-health facilities, provided such crimes meet the legal threshold for immediate arrest [24].

Deferred arrest may be executed when immediate intervention is impracticable due to safety concerns or service continuity requirements [25]. This measure permits the arrest of individuals who are clearly identified as perpetrators on the basis of video surveillance or other legally acquired digital evidence that unequivocally captures the criminal act. Such an arrest must be carried out within a strict timeframe, namely within forty-eight hours of the offence, and only after the identification of the individual in question [26].

Legislative intervention represents a necessary step in addressing the phenomenon of violence against healthcare workers. However, to date, there is a lack of empirical evidence assessing the actual effectiveness of such measures, which raises concerns about their potential insufficiency. In the absence of robust evaluations, the real impact of existing laws remains uncertain. It will therefore be essential, in the coming years, to assess—on the basis of systematic and reliable data—whether the incidence of assaults has effectively declined following these interventions. Moreover, it may become apparent that additional legislative measures are required, not only from a punitive standpoint, but also through structural reforms aimed at strengthening the healthcare system itself. A comprehensive approach that combines deterrence with organizational improvement and workforce protection is likely to be more effective in the long term.

Scholars have noted that laws addressing public health and workplace safety often carry a strong symbolic function, signaling institutional commitment without necessarily ensuring effective implementation [27]. Italy’s recent reforms risk falling into this category if not backed by robust enforcement mechanisms, adequate resources, and continuous monitoring. In fact, legal deterrence depends not only on the severity of penalties but also on the system’s capacity to enforce them, and delays in compliance, fragmented oversight, and implementation gaps could ultimately undermine the deterrent value of the legislation. Future research should therefore examine not only formal adoption but also operational effectiveness on the ground.

## 3. Profiles of Perpetrators: 2024 Data from the National Observatory on the Safety of Health and Social Work Professionals

Given the persistent resurgence of violent incidents, particularly within emergency departments, over recent years, a strategic initiative was launched in 2023 to establish a more standardized and comprehensive data collection system aimed at tracking assaults on healthcare and social-healthcare personnel. This initiative, involving the completion of specific reporting forms endorsed by the ONSEPS, was formally consolidated in 2024 [28].

The data collection system currently integrates multiple sources, including the CRGRS (Centri Regionali per la Gestione del Rischio Sanitario—Regional Health Risk Management Centers) and the ONBP (Osservatorio Nazionale sulla Sicurezza degli Esercenti le Professioni Sanitarie e Socio-sanitarie—National Observatory of Good Practices on Safety in Healthcare). Additionally, the system facilitates the structured reporting of adverse events, with voluntary submissions from healthcare professionals, including those incidents involving assaults that result in permanent injuries or fatalities, as captured in the SIMES (Sistema Informativo per il Monitoraggio degli Errori in Sanità—Information System for Monitoring Errors in Healthcare).

These reports are further augmented by annual surveys conducted by professional federations, ensuring the collection of homogeneous data across the sector. Additional sources of information include data on workplace injuries reported to INAIL (Istituto Nazionale Assicurazione contro gli Infortuni sul Lavoro—National Institute for Insurance against Accidents at Work), particularly those related to assaults resulting in absenteeism exceeding three days, as well as reports from organizations such as the Italian Red Cross. Moreover, crime statistics and law enforcement actions, as provided by the Ministry of the Interior, contribute to the comprehensive understanding of the issue.

The comprehensive data collection system promoted by ONSEPS has effectively expanded the opportunities for healthcare and social-health workers to report incidents of violence. However, as a limitation, due to the voluntary nature of the reporting process, some regions exhibit incomplete data. It should be noted that the reporting of assaults against healthcare personnel in Italy relies on voluntary disclosure and is therefore often affected by significant underreporting. As such, a higher number of recorded incidents in a given region does not necessarily reflect a higher actual prevalence of violence, but may instead indicate more robust monitoring systems or greater institutional awareness. This limitation must be carefully considered when drawing comparisons across regions. Notably, Italy’s long-standing territorial disparities in healthcare provision further complicate the interpretation of such data. Regions with stronger economies—such as Lombardy, Emilia-Romagna, and Veneto—generally benefit from greater financial resources, more advanced infrastructure, and higher staffing levels, while southern regions like Calabria, Campania, and Sicily continue to face chronic underfunding, infrastructural deficiencies, and workforce shortages [29]. These asymmetries result in measurable inequities in access and quality of care, including prolonged waiting times and elevated rates of healthcare migration, with many patients from the South traveling to the North to receive specialized treatment [30]. Moreover, the same structural disadvantages that affect service delivery in under-resourced regions—particularly in central and southern Italy—are likely to extend to the reporting process itself. In these areas, the underreporting of violence may be exacerbated by limited administrative capacity, inadequate institutional protocols, or a lack of dedicated personnel for incident documentation, thereby concealing the true extent of the problem. Despite the inherent challenges in data collection and the existence of numerous—sometimes conflicting—reports, the available data nonetheless offer a coherent and informative snapshot of the phenomenon. While imperfect, these data constitute an essential starting point for its systematic analysis and understanding. The regional disparities highlighted, indeed, may also reflect broader structural weaknesses in Italy’s decentralized healthcare governance. Regions with fewer resources often lack the infrastructure, staffing, and institutional culture necessary to promote transparent reporting practices. This asymmetry not only conceals the true prevalence of violence but may also contribute to heightened workforce stress, experiences of moral injury, and long-term retention issues among healthcare staff, especially in already under-resourced areas [31].

The available data predominantly originate from public healthcare facilities, whereas data from private institutions remain largely absent. Below is an overview of the assault statistics for selected Italian regions (Table 2) [28]. Despite the challenges associated with data collection and the presence of numerous, and at times conflicting, reports, the available data nonetheless provide a coherent and indicative representation of the phenomenon.

The overall analysis indicates that, in 2024, nearly 18,000 assaults were reported at the national level, involving approximately 22,000 healthcare workers.

Assaults against healthcare professionals occur predominantly within hospital settings, with the emergency department (ED/ER) and inpatient wards being the most frequently affected areas. However, incidents have also been reported in outpatient clinics and shared spaces. Notably, in certain regions—such as Tuscany and Lazio—a substantial number of assaults also take place within territorial healthcare facilities. While assaults are also recorded in psychiatric settings, their frequency appears comparatively lower than in emergency and inpatient contexts.

Within community-based services, territorial psychiatric facilities are the most commonly affected. Other high-risk settings include correctional institutions, territorial outpatient clinics, emergency services, and addiction treatment centers.

The majority of assaults occur during weekdays, with incidents distributed relatively evenly across time slots: mornings account for 40% of cases, afternoons 37%, and evenings and nights 23%, indicating a slight reduction in occurrences during nighttime hours.

The profile of assaulted healthcare professionals (Table 3) is predominantly female, comprising 63% of reported cases, although this trend is less marked in certain regions such as Campania and Veneto. Nurses constitute the most frequently targeted professional group (55%), followed by physicians (17.3%) and socio-health workers (9.5%). Additionally, 8% of reported cases involve non-healthcare personnel. In densely populated regions, other professional categories—such as technicians, socio-health workers, and administrative or front-office staff—also exhibit elevated rates of aggression, reaching significant levels. The age distribution of the victims is relatively uniform, spanning the entire range of the working-age population.

With regard to the aggressor profile (Table 4), the majority of incidents involve the patient themselves as the perpetrator (67%), followed by relatives or acquaintances in approximately 29% of cases. Notably, in the Calabria region, episodes of aggression perpetrated by colleagues—namely, other healthcare professionals—have also been reported. Verbal abuse constitutes the most prevalent form of violence (70%), while physical assaults account for approximately one-quarter of the cases (24%). Acts involving damage to public property are relatively rare, representing only 6% of reported incidents.

As presented in Table 5, data from professional associations indicate that the number of reported assaults exceeds 18,700, involving a total of 7320 healthcare professionals.

## 4. An Analysis of Aggression and Its Socioeconomic Implications

In light of the aforementioned limitations, the data presented raise significant concerns regarding the safety of all personnel working within healthcare settings. One is prompted to question the underlying reasons behind the occurrence of assaults, particularly why patients, who initially seek assistance, subsequently resort to violence against healthcare workers and damage medical facilities. The phenomenon of aggression is complex, as it involves individuals who first request help, only to later attack caregivers and destroy hospital property.

Another noteworthy finding is that, although the distribution of assaulted staff appears uniform across all age groups, the average age of those experiencing violence is lower than that of their colleagues who have not reported such incidents. This suggests that younger or less experienced healthcare workers may be at increased risk of assault, whereas more seasoned staff could benefit from a degree of protective effect conferred by experience. As a result, less experienced personnel emerge as a particularly vulnerable subgroup within the healthcare workforce [32].

Our data also confirm that female nurses are disproportionately affected by verbal abuse in healthcare settings compared to their male counterparts. This finding aligns with international evidence and is consistently reported across the literature. However, while frequently acknowledged, it is rarely examined through a critical lens that interrogates the gendered power dynamics and occupational hierarchies underpinning such disparities. Female nurses occupy a professional position that is historically and structurally subordinate to that of physicians and hospital administrators—a configuration that reflects broader societal patterns of gendered labor stratification [33]. Within this context, verbal abuse cannot be reduced to isolated interpersonal incidents; rather, it should be understood as a manifestation of deeper systemic inequities. The feminization of nursing is closely tied to diminished professional authority, lower remuneration, and constrained access to institutional decision-making processes [34]. These intersecting vulnerabilities render female nurses more susceptible to both vertical violence (e.g., from physicians or supervisors) and horizontal violence (e.g., from patients or colleagues), particularly in settings where rigid hierarchies and unchallenged gender biases persist [35]. In addition, entrenched cultural expectations surrounding emotional labor and caregiving—roles traditionally ascribed to women—can further obscure the recognition and reporting of abuse. Such behaviors are often internalized or normalized as inherent to the nursing profession, thus reinforcing a cycle of invisibilized harm [36]. These findings should be interpreted within the broader context of gendered occupational hierarchies in healthcare systems, since female nurses, often positioned at the intersection of professional subordination and societal caregiving expectations, face compounded vulnerability to verbal abuse. This disproportionate targeting reflects the structural undervaluation of nursing work and entrenched power asymmetries between nurses, physicians, and patients. Moreover, in highly hierarchical healthcare environments, these dynamics can manifest as both horizontal and vertical violence, reinforcing cycles of psychological harm and underreporting [37].

There is also a marked difference in the distribution of assaults between the public and private healthcare sectors, which varies according to the specific profession. For instance, physicians, midwives, and social workers face higher risks in the public sector, whereas veterinarians encounter a greater risk in private settings (70%). The settings most prone to violent incidents also vary. For physicians, the most hazardous locations include public outpatient clinics and inpatient wards, while pharmacists face higher risks in pharmacies and parapharmacies. Interestingly, physical therapists appear to face a uniform risk across different settings [28].

Violence against physicians and healthcare workers represents a pressing and multifaceted public health issue, whose root causes span individual, organizational, and structural dimensions. At the individual level, patient-related factors such as untreated psychiatric illness, substance abuse, cognitive impairment, and frustration due to unmet expectations are well-established triggers of aggression in healthcare settings [38]. These risks are particularly pronounced in emergency departments, where the clinical context often involves acute distress and impaired patient self-regulation. In recent years, the prevalence of untreated psychiatric conditions—especially among patients seeking emergency care—has further compounded the incidence of violence, heightening the vulnerability of both healthcare staff and other patients. Importantly, those patients whose access to timely care is obstructed by violent incidents may become indirect victims of such aggression themselves [39].

On the organizational level, several systemic issues have intensified the risk of aggression toward healthcare personnel. Budgetary constraints in the healthcare sector have had far-reaching effects across the entire service chain, resulting in staff shortages, reduced hospital bed capacity, and shorter average hospital stays. These resource limitations have coincided with an increase in patient volumes and a decline in the perceived quality of care, collectively fostering frustration among both patients and healthcare workers [40]. Contributing factors such as prolonged waiting times, poor communication, and reduced interpersonal interaction due to high patient turnover further exacerbate dissatisfaction [41]. These dynamics create a fertile ground for conflict, particularly when patients feel neglected, dismissed, or overwhelmed by the complexity of healthcare procedures.

From a broader socio-institutional perspective, the normalization of violence within healthcare settings—especially in high-pressure environments like emergency departments—reflects a deeper cultural malaise. The erosion of trust in medical institutions, coupled with a lack of legal safeguards and institutional accountability, has created a context in which violence is frequently underreported and, in some settings, tacitly tolerated [42] Healthcare workers may refrain from reporting incidents due to fear of retaliation, perceived futility, or a widespread cultural acceptance of violence as an inevitable aspect of their professional role—phenomena often interpreted through the lens of institutional anomie [43]. Moreover, structural inequalities such as low health literacy, limited access to healthcare services, and pronounced socioeconomic disparities contribute indirectly by fostering resentment, disempowerment, and adversarial attitudes toward the healthcare system [44].

Physicians and other healthcare workers are not mere passive targets; they are integral to the functioning of the healthcare system, and their safety is vital for the well-being of both patients and professionals alike [45]. In addition, the phenomenon of violence against healthcare personnel imposes significant economic burdens on healthcare systems. For instance, the management of both physical and psychological injuries sustained by victims of aggression, the loss of workdays, disruptions in services, the temporary or permanent replacement of affected professionals, as well as the recognition and compensation of injuries through the INAIL, all contribute to substantial costs. Additionally, there are legal expenses, damage to facilities and property, and indirect costs associated with violence prevention measures. The diminished quality of care, increased risk of medical errors, and higher insurance premiums further exacerbate the financial strain on healthcare systems [46].

## 5. Strategies to Address the Phenomenon of Violence

In recent years, healthcare personnel have become increasingly aware of their heightened exposure to dangerous situations while performing their professional duties. Consequently, preventive and defensive measures to safeguard healthcare professionals have been the subject of extensive study and implementation [47,48].

At both the national and regional levels, actions have been undertaken to address this issue, focusing on the collection and analysis of violence data, as well as identifying regional variances in the prevalence and nature of assaults. The measures undertaken can be broadly categorized into three domains: prevention, protection, and prosecution. In terms of prevention, one of the earliest responses was the implementation of a national initiative aimed at strengthening training programs and preventive strategies. This included the introduction of regional and national training programs aimed at addressing the ongoing issue of violence against healthcare workers [49,50]. In 2025, the government formalized this approach with a memorandum of understanding between the Ministry of Health, the Italian Federation of Health and Hospital Companies, and Federsanità, aimed at combating aggression within healthcare settings. This agreement focuses on developing and disseminating uniform training courses for health and social-health personnel across the country, incorporating initiatives to raise awareness of the critical importance of the work performed by healthcare workers and related professionals [51].

However, the measures implemented at the institutional level have been somewhat varied. For example, at the Umberto I University Hospital in Rome, self-defense courses were introduced to foster a culture of zero tolerance for violence against healthcare workers [52] The courses, which were first held on the National Day for Education and Prevention Against Violence Against Health Workers on March 12, focus on both theoretical and practical components [53]. Theoretical instruction involves identifying early signs of aggression, while the practical sessions provide training on specific self-defense techniques.

An additional preventive measure involves reaffirming the fundamental principle underpinning all such efforts: the cultivation of mutual respect for the work of others and the recognition that, without the support and collaboration of healthcare professionals, the delivery of effective care would be critically undermined [54].

The focus should be on emphasizing the value of the work done by doctors and nurses, without attributing blame for the inefficiencies of the public healthcare system. As Peppe Dell’Acqua eloquently stated [55], the SSN grants every citizen the right to access healthcare with dignity. Within this system, healthcare workers provide more than just medical treatment—they offer something invaluable: the recognition of others as equals, with a respect that transcends monetary value. This act of care is a delicate and voluntary one, not a mere obligation [56].

To this end, public awareness campaigns—targeting both educational and re-educational levels—may serve as effective tools. For instance, the National Medical Association has promoted such efforts through the dissemination of posters and messages via social media platforms.

Protective measures are progressively being implemented across various healthcare settings in Italy, ranging from hospitals to individual medical practices.

A notable innovation was introduced at the Hospital of Bologna, where a partnership between the Bologna Police Headquarters and the leadership of the city’s three main health institutions—Ausl, Policlinico Sant’Orsola, and Istituto Ortopedico Rizzoli—led to the installation of thirty ‘red buttons’ in critical areas of the hospitals, particularly in emergency rooms. These buttons allow healthcare personnel to immediately alert security staff and police if they experience an assault. Upon activation, the system triggers sound alerts and notifies hospital security, the police station, and the local police headquarters, with the precise location of the incident, enabling a rapid and effective response [57]. This system was first utilized on 20 March 2025, shortly after its inauguration [58]. Another example of institutional responses includes a situation in a small Cilento village (in the Campania region), where a general practitioner, faced with persistent verbal aggression from patients, was forced to employ private security services [59].

Finally, with regard to prosecution, there is growing discussion around the adoption of prompt and supplementary legal measures aimed at preventing recidivism among perpetrators of violence. In response to the alarming rise in violent assaults targeting healthcare personnel in Sicily, a recent multidisciplinary summit—bringing together medical professionals, healthcare administrators, and legal scholars—culminated in a unified and urgent appeal: “No more impunity. Access to care must be denied to those who attack those who provide it”. The proposal currently under discussion entails the introduction of a so-called Health DASPO (Divieto di Accedere alle manifestazioni Sportive—Prohibition Order from Attending Sports Events), a regulatory public order mechanism aimed at restricting access to institutional facilities for individuals deemed a threat on the basis of violent or dangerous conduct. Specifically, the measure would impose a suspension of up to three years from access to non-urgent and elective healthcare services for individuals convicted of perpetrating violence against healthcare workers or committing offences against healthcare infrastructure [60].

These examples illustrate a growing commitment to the implementation of initiatives designed to counter aggression against healthcare professionals. Nevertheless, it is crucial that such proposed interventions are supported by adequate resources and subjected to ongoing evaluation, allowing for necessary adjustments and further development over time. In this context, longitudinal studies are essential to assess the enduring impact of legislative measures, particularly with respect to their deterrent capacity and influence on behavioral change. Furthermore, qualitative inquiries into the lived experiences and perceived safety of healthcare professionals in the aftermath of legislative reforms would provide valuable insights into the real-world applicability and consequences of such measures. These lines of investigation hold the potential to inform the refinement of existing policies and support the development of more adaptive, context-sensitive, and evidence-based strategies [56].

Moving from principles to practice, several actionable recommendations emerge for the Italian context. First, mandatory de-escalation and communication training programs should be standardized nationwide and incorporated into continuing education requirements. Second, the creation of integrated digital platforms for reporting incidents—accessible across all healthcare facilities—could improve data quality and encourage transparency. Third, victim support protocols should include psychological counseling, legal assistance, and structured follow-up, modeled after existing programs in Scandinavian healthcare systems. Fourth, the Ministry of Health should coordinate efforts to harmonize regional reporting standards, thereby reducing territorial disparities and enabling more consistent interventions. Finally, public education campaigns co-designed with community groups could promote respect for healthcare workers and rebuild trust, emphasizing the shared vulnerability and dignity of all individuals within the healthcare space.

## 6. Conclusions

Suffering should never be accompanied by exclusion. Being a patient or a healthcare professional does not strip away humanity or rights. In Italy, we are fortunate to have one of the most respectful healthcare laws in the world, which ensures that individuals remain citizens, regardless of their role as patients, and that healthcare workers retain their dignity despite facing violence. The fabric that binds our SSN is this shared humanity, a thread that connects both the caregivers and those they serve. The healthcare setting must return to being a sanctuary of care, not one of violence and aggression.

There are many reasons behind the violent behavior observed in these spaces of human interaction, but perhaps the key lies in how we respond to it. Healthcare facilities must adopt a firm stance against any form of violence and stop viewing assault as a mere occupational hazard, as has historically been the case. Healthcare workers should not be seen as scapegoats for systemic issues or delays within the SSN, which often leads to the erosion of morale and the diminishing of genuine authority.

There is a pressing need for multi-stakeholder collaboration that actively engages policymakers, healthcare institutions, law enforcement agencies, and civil society organizations. Such a coordinated and comprehensive approach is essential to effectively address and mitigate violence against healthcare professionals, ensuring that preventive, protective, and prosecutorial measures are harmonized and mutually reinforcing. This collective effort would facilitate the development of integrated strategies that are more responsive to the complex dynamics underlying aggression in healthcare settings.

A first step forward is targeted outreach aimed at restoring the broken trust between the public and healthcare professionals. The time has come for change, for active listening, and for the restoration of care—not only for the patient but also for the caregiver. This dual focus is crucial to ensure the future of our healthcare system, where respect and compassion prevail above all.

## Figures and Tables

**Table 1:** Main legislative measures to hinder violence against health workers in Italy, France, the UK, and the USA.

Country	Legal Reform Title	Key Measures
Italy	Law 113/2020, Law 171/2024	Ex officio prosecution, mandatory arrest, Health DASPO
France	Loi n° 2021-502	Harsher penalties, public awareness campaigns
UK	Assaults on Emergency Workers Act 2018	Higher sentences, protection for broader worker categories
USA	Various state laws	Mandatory prevention plans, staff training requirements

**Table 2:** Frequency and territorial distribution of assaults.

Italian Region	Number of Assaults	On Territory	Hospital	Inpatient Area	ED/ER	Psychiatry	Other
Abruzzo	118	5	113	44	40	18	11
Basilicata	26	5	21	8	2	0	11
Calabria	49	6	43	3	24	6	10
Campania	281	56	225	50	138	15	22
Emilia-Romagna	2684	662	2022	853	552	238	379
Friuli-Venezia Giulia	571	185	386	157	83	53	93
Latium	1156	567	589	106	263	101	119
Liguria	760	130	630	170	172	54	234
Lombardy	4604	690	3914	1590	1184	69	1071
Marche	251	86	165	28	95	27	15
Molise	46	7	39	2	12	25	0
Piedmont	1370	88	1282	352	473	280	177
Bolzano	187	17	170	24	96	35	15
Trento	250	78	172	46	70	46	10
Apulia	234	91	143	45	54	19	25
Sardinia	264	57	207	62	43	78	24
Sicily	200	10	190	60	96	17	17
Tuscany	1965	923	1042	275	372	156	239
Umbria	207	83	124	52	39	11	22
Aosta Valley	34	5	29	11	2	14	2
Veneto	2595	144	2451	674	662	599	516
Total	17,852	3895	13,957	4612	4472	1861	3012

**Table 3:** Demographics and professional categories of victims.

Italian Region	Total Operators	Males	Females	Physicians	Nurses	Other
Abruzzo	123	47	78	31	74	18
Basilicata	26	8	18	4	18	4
Calabria	63	30	33	19	34	10
Campania	471	259	212	119	221	131
Emilia-Romagna	2878	749	2098	407	1733	738
Friuli-Venezia Giulia	794	202	592	126	419	249
Latium	1840	588	1252	419	1159	262
Liguria	1230	354	876	128	668	434
Lombardy	5690	2003	3447	1243	3405	1042
Marche	251	166	85	15	174	62
Molise	61	19	27	18	25	18
Piedmont	2035	603	1432	325	1238	472
Bolzano	221	64	163	18	154	49
Trento	464	21	52	60	201	203
Apulia	239	85	150	80	85	74
Sardinia	264	101	163	48	139	77
Sicily	308	93	119	46	106	156
Tuscany	2197	553	1575	286	1136	775
Umbria	262	80	182	65	153	44
Aosta Valley	34	7	27	4	20	10
Veneto	2598	1713	882	312	1538	748
Total	22,049	7745	13,463	3773	12,700	5576

**Table 4:** Profiles of aggressors and typologies of aggression.

Italian Region	Physical Aggression	Verbal Aggression	Damage to Property	User/Patient	Relative/Caregiver	Outsider
Abruzzo	26	93	16	82	4	3
Basilicata	3	23	0	12	6	0
Calabria	35	28	5	26	19	4
Campania	148	231	38	166	106	8
Emilia-Romagna	710	2219	335	1778	749	91
Friuli-Venezia Giulia	168	474	34	408	152	9
Latium	594	1690	141	782	291	83
Liguria	208	642	84	439	200	55
Lombardy	1251	3691	368	3182	1202	123
Marche	85	157	9	165	65	5
Molise	17	26	8	31	6	0
Piedmont	389	1375	144	1091	767	61
Bolzano	73	136	0	163	20	4
Trento	119	173	61	207	41	4
Apulia	82	205	26	145	69	18
Sardinia	102	179	3	177	52	6
Sicily	104	131	5	126	84	4
Tuscany	494	1847	95	1248	553	100
Umbria	43	187	14	133	69	5
Aosta Valley	15	25	3	30	10	3
Veneto	836	2430	0	1747	782	66

**Table 5:** Occupational categories of involved workers based on association reports.

National Federation	Practitioners Involved	Total Number of Assaults
Social workers	1.055	6.867
Biologists	24	97
Chemists and physicists	9	9
Pharmacists	1.971	>4.000
Physiotherapists	438	512
Nurses	248	1.543
Physicians and Dentists	2.006	2.006
Veterinary surgeons	70	178
Midwives	779	1.364
Psychologists	295	714
Health care technicians	335	1481
Total	7.230	18,771

## Data Availability

The original contributions presented in this study are included in the article. Further inquiries can be directed to the corresponding author.
